# Neutron and hard X-ray diffraction studies of the isothermal transformation kinetics in the research reactor fuel candidate U–8 wt%Mo

**DOI:** 10.1107/S1600576716005744

**Published:** 2016-05-16

**Authors:** Steffen Säubert, Rainer Jungwirth, Tobias Zweifel, Michael Hofmann, Markus Hoelzel, Winfried Petry

**Affiliations:** a Heinz Maier-Leibnitz Zentrum (MLZ), Technische Universität München, Lichtenbergstrasse 1, D-85748 Garching, Germany; b Physik-Department, Technische Universität München, James-Franck-Strasse 1, D-85748 Garching, Germany

**Keywords:** uranium, nuclear fuels, uranium–molybdenum alloys, isothermal transformation kinetics, X-ray diffraction, neutron diffraction

## Abstract

Exposing uranium–molybdenum alloys (UMo) retained in the γ phase to elevated temperatures leads to transformation reactions during which the γ-UMo phase decomposes into the thermal equilibrium phases, *i.e.* U_2_Mo and α-U. Since α-U is not suitable for a nuclear fuel exposed to high burn-up, it is necessary to retain the γ-UMo phase during the production process of the fuel elements for modern high-performance research reactors. The present work deals with the isothermal transformation kinetics in U–8 wt%Mo alloys for temperatures between 673 and 798 K and annealing durations of up to 48 h. Annealed samples were examined at room temperature using either X-ray or neutron diffraction to determine the phase composition after thermal treatment, and *in situ* annealing studies disclosed the onset of phase decomposition. While for temperatures of 698 and 673 K the start of decomposition is delayed, for higher temperatures the first signs of transformation are already observable within 3 h of annealing. The typical C-shaped curves in a time–temperature–transformation (TTT) diagram for both the start and the end of phase decomposition could be determined in the observed temperature regime. Therefore, a revised TTT diagram for U–8 wt%Mo between 673 and 798 K and annealing durations of up to 48 h is proposed.

## Abstract 2

The nuclear fuel candidate U–8 wt%Mo has been investigated by means of neutron and hard X-ray diffraction, revealing the isothermal transformation kinetics of the high-temperature γ-UMo phase into its thermal equilibrium microstructures.

## 1. Introduction   

In order to reduce the amount of highly enriched uranium (HEU) fuel in the civilian nuclear fuel cycle, efforts are being made to develop a fuel with a higher uranium density which would allow the conversion of research and test reactors from HEU to lower enriched uranium (LEU) while maintaining an equivalent neutron flux and quality. Since uranium compounds like U_3_Si_2_ and UAl_*x*_ do not provide the uranium density which is required to convert high-performance research reactors, a new fuel has to be developed. Pure metallic uranium, which would offer the highest uranium density possible, is known to show unfavourable behaviour during irradiation to high burn-up (Frost, 1994 ▸; Hofman & Walters, 1994 ▸; Paine & Kittel, 1956 ▸; Rest *et al.*, 1998 ▸). Only the γ phase of uranium has adequate properties to be used as a nuclear fuel (Frost, 1994 ▸). Both UMo and UZrNb alloys retain the uranium γ phase in a metastable state at room temperature and have a sufficient uranium density.

However, compared with UMo the ternary alloy UZrNb has a lower uranium density and γ stability and hence shows a poorer performance in terms of predictable swelling behaviour and high fission rate during both annealing and in-pile irradiation experiments under research reactor conditions, *i.e.* high burn-up and high temperature (Meyer *et al.*, 2014 ▸; Snelgrove *et al.*, 1996 ▸). Therefore the UMo alloy, well known since the 1950s, is currently the subject of renewed interest among the international research reactor fuel-developing community. The addition of 7–10 wt% of Mo to the U is the best compromise between a high uranium density and a good γ stability of the UMo alloy (Hofman *et al.*, 1998 ▸).

Nevertheless, the high temperatures to which the fuel element is exposed during the manufacturing process may lead to a decomposition of the UMo γ phase into its thermal equilibrium microstructures, *i.e.* α-U and U_2_Mo. Although it has been shown that the decomposition is reversed during in-pile and heavy-ion irradiation (Bleiberg *et al.*, 1956 ▸; Konobeevskii *et al.*, 1967 ▸; Jungwirth, 2011 ▸), it is preferable to avoid it during fuel plate production. Therefore, the precise kinetics of the γ-UMo phase decomposition need to be understood. Since the available time–temperature–transformation (TTT) diagrams are based on data from the 1950s and 1960s, a new study applying more modern techniques seemed to be advisable.

Therefore, both neutron and X-ray diffraction studies at room temperature were performed on annealed samples in order to obtain detailed crystallographic information on the state of decomposition as a function of time and temperature. Additionally, *in situ* annealing studies with neutron diffraction were used for the investigation of peak-growth behaviour and hence the transformation kinetics of single phases.

## 2. Sample preparation   

All samples analysed in this work originated from the same U–8 wt%Mo ingot provided by the AREVA-CERCA company (Romans, France). The samples were cut down from the ingot, melted in an electric arc furnace and cast into a cylindrical shape. After that, all the samples were homogenized at 1173 K for 48 h *in vacuo* to minimize oxidation and then water quenched to room temperature. This ensured that only the γ-UMo phase was captured in all the specimens before heat treatment. The presence of a single γ-UMo phase was verified by neutron diffraction analysis on one sample which was prepared as described.

### 2.1. Heat treatment   

Depending on the annealing duration and temperature, the decomposition of the γ-UMo phase is captured at different stages of transformation. Therefore, after homogenization, the samples were annealed at different temperatures between 673 and 773 K and with annealing times of 3, 6, 16, 24 or 48 h in order to obtain a reasonable grid of measurement points with various stages of γ-UMo phase decomposition. During the heat treatment the samples were again kept *in vacuo* in order to minimize oxidation.

## 3. Crystallographic phase analysis   

Neutron diffraction experiments were performed at the Forschungs-Neutronenquelle Heinz Maier-Leibnitz (FRM II) (Garching, Germany). The phase composition in the pre-annealed samples was studied on the high-resolution structure powder diffractometer SPODI at the FRM II (Hoelzel *et al.*, 2012 ▸). For the experiment, a germanium monochromator Ge(551) was chosen, together with a take-off angle of 155° and a 5 m distance to the sample. Measurement of the NIST Si-640c standard along with a Rietveld refinement of the diffraction pattern determined the wavelength to be λ = 1.548 Å. In total, 12 samples were analysed, which were heat treated according to Table 1 ▸. A 2θ step width between 0.05 and 0.1° was chosen, along with scan times between 6 and 8 h. Data were collected in the angular range 1.0–151.8° in 2θ, *i.e.* 0.006–0.626 Å^−1^ in sin(θ)/λ.

X-ray diffraction measurements were carried out at the Deutsches Elektronen-Synchrotron (DESY) (Hamburg, Germany). The phase composition in the pre-annealed samples was studied on the high-energy materials science beamline P07 (HEMS) of the Positron–Electron Tandem Ring Accelerator III (PETRA III) at DESY (Schell *et al.*, 2014 ▸). With an energy of *E* = 100 keV the wavelength is calculated to be λ = 0.124 Å, which was confirmed by measurement of the NIST LaB_6_-660a standard. In total, seven samples were analysed, which were heat treated before measurement according to Table 2 ▸. Data were collected in the angular range 0.0025–7.6400° in 2θ, *i.e.* 0.0004–0.537 Å^−1^ in sin(θ)/λ.

### 3.1. Data analysis   

**Figure 1:**
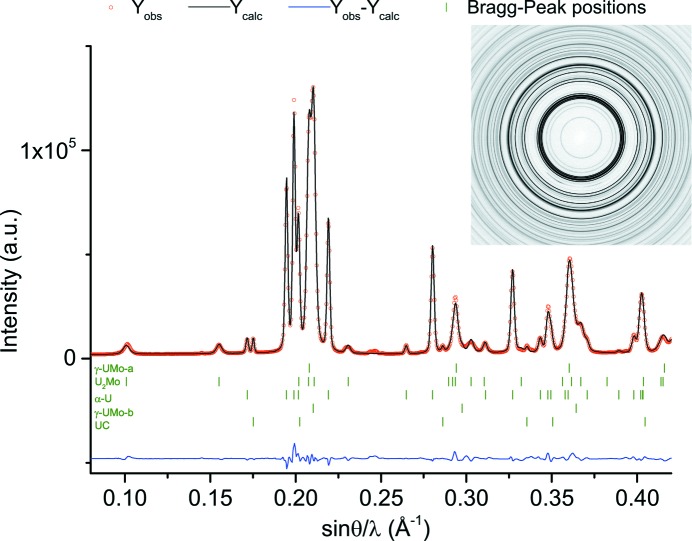
The X-ray diffraction pattern for the sample A-748K16h with the diffraction image (inset). Red circles indicate the measured data, green dashes the Bragg peak positions, the black line the calculated pattern, and the blue line the difference between the calculated pattern and the measured data.

Diffraction data collected by either neutron or X-ray diffraction were analysed using the Rietveld refinement method (Rietveld, 1969 ▸) and the *FULLPROF* software package (Rodríguez-Carvajal, 1993 ▸). Five phases were included in the refinement process. Four of them describe the UMo-phases: γ-UMo-a (space group 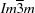), γ-UMo-b (space group 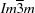), α-U (space group *Cmcm*) and U_2_Mo (space group *I*4/*mmm*). γ-UMo-a represents the initial γ phase and γ-UMo-b a molybdenum-enriched γ phase which precipitates during the phase decomposition reactions. Hence, the latter phase has smaller lattice parameters (Dwight, 1960 ▸). α-U and U_2_Mo are the final products of decomposition, and the α phase can also be in the distorted states α′-U or α′′-U, depending on the reaction temperature. α′-U is a distorted α phase characterized by a contraction of the *b* parameter together with an expansion of the parameters *a* and *c*, whereas α′′-U is a further distortion of the parameters *a*, *b* and *c* along with a phase change from an orthorhombic to a monoclinic structure. An enrichment in Mo leads to a distortion of the lattice and hence the formation of the phases α′-U and α′′-U (Lehmann & Hills, 1960 ▸; Orlov & Teplinskaya, 1999 ▸; Stewart & Williams, 1966 ▸). Owing to the inclusion of carbide and nitride during the production process of the material by the AREVA-CERCA company, one other phase was added. Since UC and UN have the same space group, *i.e.* 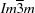, and very similar lattice parameters, only one phase representing both of them was included and named UC. Fig. 1 ▸ shows the Bragg peak position of each included phase, along with the results of the Rietveld refinement and an example diffraction pattern.

For the refinement, pseudo-Voigt functions were chosen to fit the Bragg peak shapes. The background was described by selected background points and a linear interpolation between these, rather than by mathematical functions. The analysis included the refinement of scale factors, lattice parameters and peak-shape parameters. Moreover, to improve the quality of the fit, the background points were refined as well.

### 3.2. Crystallographic composition   

Example Rietveld refined diffraction patterns for XRD and neutron diffraction are shown in Figs. 1 ▸ and 2 ▸, respectively. Both diffraction patterns were taken after annealing at 748 K for either 16 or 48 h. After 16 h of annealing at this temperature the decomposition is already in an advanced stage. Between 16 and 48 h of annealing, most of the remaining γ-UMo is decomposed into the equilibrium microstructures, *i.e.* α-U and U_2_Mo, as can be seen from the pattern taken after 48 h.

## 4. *In situ* annealing studies   

Diffraction studies of γ-UMo samples during *in situ* annealing were performed on the materials science diffractometer STRESS-SPEC at the FRM II (Hofmann *et al.*, 2006 ▸). For the experiment, a germanium monochromator Ge(311) and a 1.065 m distance to the sample were chosen. Measurement of the NIST Si-640c standard showed a wavelength of λ = 1.914 Å. Data were collected in the angular range of 37.8–56.7° in 2θ, *i.e.* 0.169–0.248 Å−1 in sin(θ)/λ. This angular range was chosen because the most distinctive peaks of each individual phase are located in this range. Therefore, it is theoretically possible to observe the following peaks (underlined peaks overlap with other peaks; bold peaks are unaffected by other peaks; roman peaks are prohibited): 
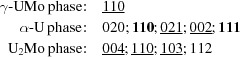
Diffraction patterns were collected every 5 min while annealing the samples in a high-temperature furnace evacuated to a high vacuum (∼10^−6^ mbar; 1 bar = 100 kPa). The temperature was monitored using a C-type thermocouple pressed on top of the sample, separated only by a thin vanadium foil. Besides a second C-type thermocouple next to the first one as a reference, the reliability of the temperature control was verified by observing the structural phase transition in a lead(II) titanate (PbTiO_3_) standard. The samples were heated at a rate of 10 K min^−1^, which allowed the sample to adapt to the set temperature in a controlled way. Because of this high heating rate, the samples spent only a short time in the temperature regime where decomposition starts early, and hence there are only negligible effects on the transformation process. Aluminium windows around the sample position allowed the neutrons to penetrate the experimental setup easily. In total, five specimens were investigated as shown in Table 3 ▸.

### 4.1. Data analysis   

**Figure 3:**
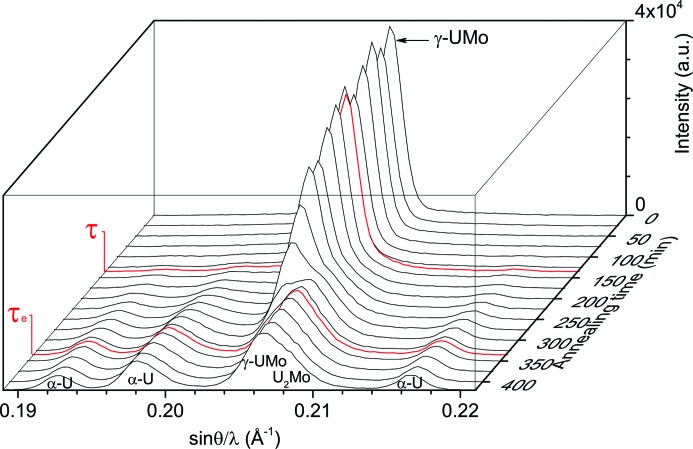
A waterfall chart of the γ-UMo phase decomposition as a function of annealing time at 773 K (sample A-773Kinsitu). The diffraction patterns for τ = 10% and τ_e_ = 90% are highlighted in red.

Consecutive collected diffraction patterns (an example is shown in Fig. 3 ▸) were analysed using *StressTextureCalculator* (Randau *et al.*, 2011 ▸). This software processes all diffraction patterns sequentially. The growth of a single peak was determined by observing the sum of intensities over an angular range as a function of time. Thus, the angular range is the same for all diffraction patterns of one sequential measurement. This method was preferred over fitting each individual peak, since the fit was not good for very small peaks, *i.e.* the beginning of peak growth.

The obtained curves of peak growth with time were then analysed with a modified Avrami equation (Avrami, 1939 ▸, 1940 ▸, 1941 ▸):
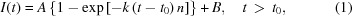
where the parameters *n* and *k* describe the nucleation and growth process [for a detailed description of the nucleation and growth kinetics, see Christian (2002 ▸) and Avrami (1939 ▸, 1940 ▸, 1941 ▸)], *A* is a scale factor for the intensity, *B* describes the background intensity, and *t*_0_, with a lower limit of *t*_0_ = 0, is introduced in order to describe peak-growth curves which do not start at *t* = 0. The peak intensity as a function of annealing time could only be described quantitatively for the α-U phase since, because of overlapping peaks, the quantitative extraction of the intensities of the other phases, *i.e.* the growth of U_2_Mo and the decrease of γ-UMo, was not feasible.

**Figure 4:**
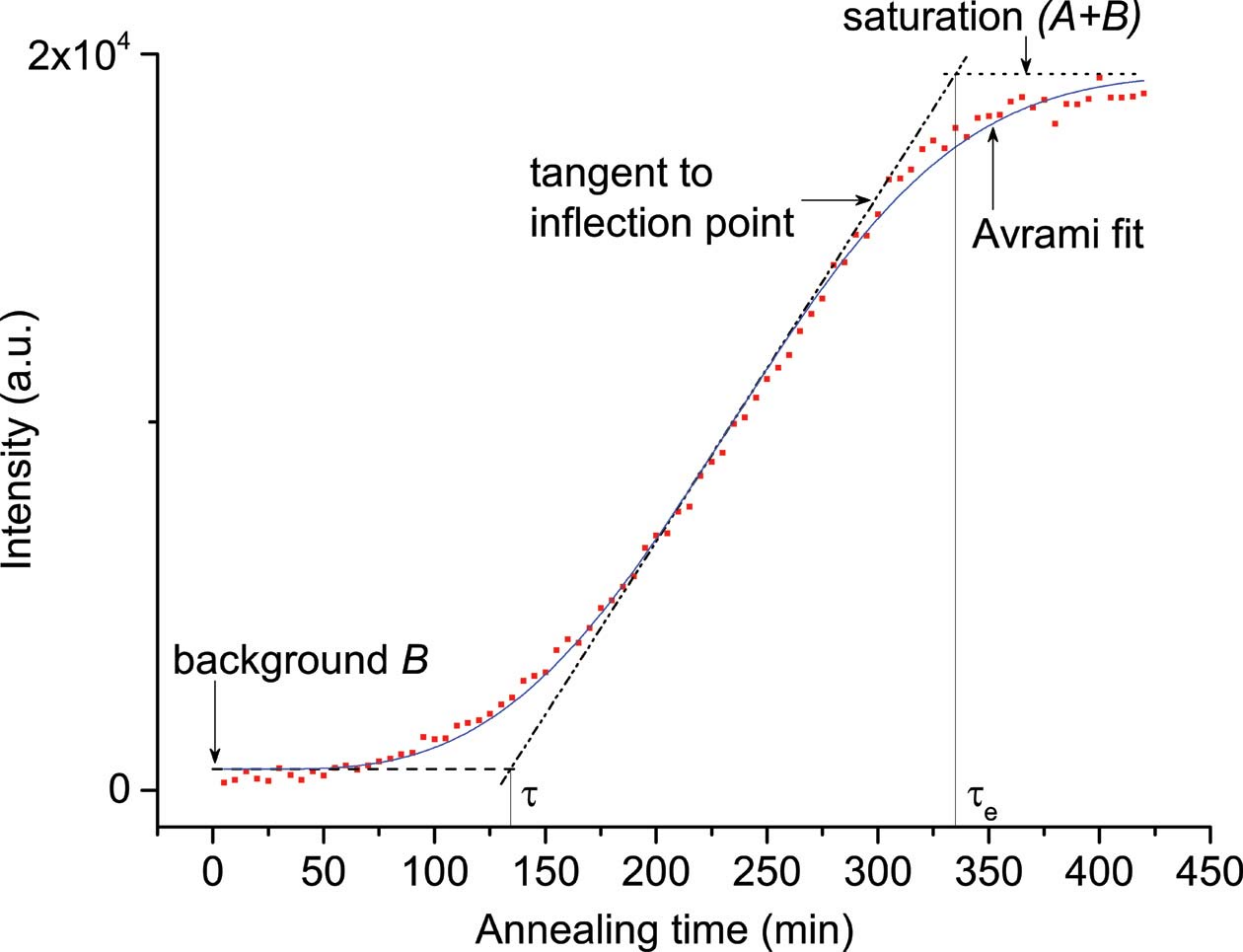
Peak growth and the Avrami curve for the 110-α-U phase during annealing at 773 K (sample A-773Kinsitu).

Peak-growth curves were obtained for α-U at the different temperatures shown in Table 3 ▸. On the basis of the information obtained from these measurements, Avrami curves were calculated in order to determine the beginning and end of α-U phase growth as a function of annealing temperature. An example of this peak growth is shown in Fig. 4 ▸.

With the obtained fit parameters, definitions of the times τ and τ_e_ were found by taking the intersections of the tangent to the inflection point with the background and saturation, respectively. Moreover, with the Avrami equation any fraction of the transformed phase can be calculated at any time. Similar calculations were made for each individual measurement. The most significant durations for transformed α-U as a function of annealing temperature are shown in Table 4 ▸.

The diffraction patterns collected in the angular range of 0.169–0.248 Å^−1^ in sin(θ)/λ showed that, because of the overlap of the peaks 110-γ-UMo, 110-U_2_Mo and 103-U_2_Mo, it was not possible to observe the correct peak intensity increase and decrease of these peaks. It should be noted that a second γ-UMo phase was not included in the evaluation since it is not possible to distinguish a second γ-UMo phase at about the same position as the first one. Also, the peaks 021-α-U, 002-α-U and 004-U_2_Mo overlap each other. Since all of them increase with time, the start of phase decomposition could be observed. Owing to the above-mentioned short-term measurements and the small angular range, the exact content of α-U could not be calculated *via* Rietveld refinement. Therefore, it was not possible to determine the intensities of the single phases 021-α-U and 002-α-U and hence an estimated intensity for 004-U_2_Mo.

### 4.2. α-U phase-growth kinetics   

The beginning of α-U phase growth is also the beginning of phase decomposition, since α-U is, together with U_2_Mo, the first product of the intermediate- and high-temperature reactions (Repas *et al.*, 1964 ▸; Van Thyne & McPherson, 1957 ▸; Blake & Hehemann, 1976 ▸). Information on the beginning and end of peak growth is obtained by observing the peak intensities as a function of time. Thus, the most promising peaks are 110-α-U and 111-α-U, because these are the only peaks which do not overlap with any other. Also observable are the peaks 021- and 002-α-U, which are seen as one peak since it is not possible to distinguish between them. Both peaks grow equally and hence the result for the start of phase decomposition, obtained by observing the peak growths together, is correct.

## 5. Results   

While the *in situ* annealing measurements performed here determined the onset of phase decomposition, as well as giving detailed information on α-U phase growth, diffraction patterns at different points in time and temperature were used to derive the crystallographic phase composition at these points. Hence, *in situ* measurements gave information on the time a phase started to decompose, but not on the exact crystallographic composition as a function of time and temperature, because of the limited coverage in reciprocal space. Crystallographic phase analysis, on the other hand, delivered detailed information on the phase composition in the samples at a certain time and temperature, but none about how this state was reached. Therefore, a complementary consideration of both methods is required in order to obtain a significant isothermal transformation diagram for U–8 wt%Mo.

### 5.1. Phase decomposition measurements during *in situ* annealing   

The data show the strong temperature dependence of the onset of phase decomposition and the transformation itself. As expected, the transformation starts earlier for higher temperatures, while it is delayed and much slower for lower temperatures. Hence, the transformation is divided into high-temperature reactions (above 748 K) and intermediate-temperature reactions (698–748 K).

#### 5.1.1. High-temperature reactions   

In order to investigate the early beginning of phase decomposition, *in situ* annealing studies were performed at 798 K. We expected to observe the phase transformation in less than 2 h and hence the total annealing time was set to 4.5 h. Diffraction patterns were collected every 5 min. Even though the total annealing time of 4.5 h was too short to observe the end of peak growth for α-U, a full Avrami fit was possible and allowed us to derive not only τ but also τ_e_: τ ≃ 100 min and τ_e_ ≃ 275 min. Another specimen was annealed for 7 h at 773 K and diffraction patterns were collected every 5 min. The Avrami fit delivers τ ≃ 135 min and τ_e_ ≃ 335 min.

By regarding the peak-growth curves (an example curve of α-U peak growth is shown in Fig. 4 ▸), it becomes apparent that the nucleation period τ does not describe the very beginning of phase decomposition: it is obvious that there is already some transformed material before the nucleation period is reached. This is a result of the definition of τ, using the inter­section of the linear part with the background. A comparison of the data obtained during *in situ* annealing and the data obtained by crystallographic phase analysis will be conducted in §5.3, and the definition of the start of phase decomposition will be discussed there in more detail.

**Figure 5:**
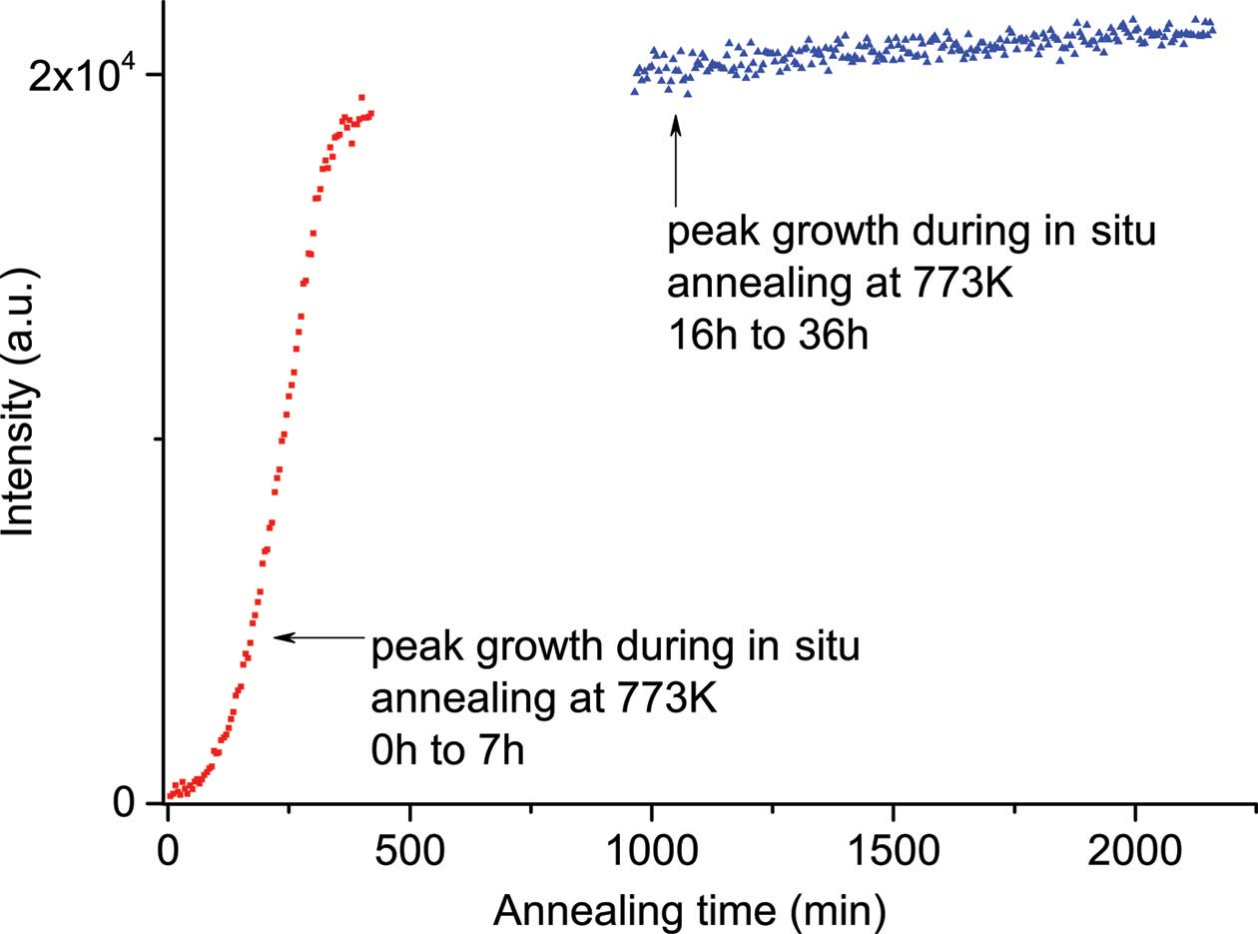
Peak growth and Avrami curves for the 110-α-U phase during annealing at 773 K (samples A-773Kinsitu and A-773K16h) from 0 to 7 h and on a pre-annealed sample from 16 to 36 h.

Sample A-773K16h, which has already been measured with XRD, was annealed *in situ* for another 20 h at 773 K and diffraction patterns were collected every 5 min. Fig. 5 ▸ shows the 110-α-U peak intensities for the measurements from 16 to 36 h, together with the measurements from 0 to 6 h. It can easily be seen that the saturation described by the Avrami curve does not exactly define the end of peak growth. After the fast growth described by the Avrami curve, the peak keeps on growing slowly with a linear-like increase. According to this, the Avrami theory does not describe peak growth in UMo exactly, but it does describe the fast growth, up to the point where the linear-like growth starts. For further discussions the Avrami curve always describes this fast growth at the beginning of phase decomposition rather than the total peak growth. This will be of great importance for the discussion and comparison of data obtained from diffraction patterns and peak-growth curves.

#### 5.1.2. Intermediate-temperature reactions   

One sample was annealed for 10 h at 723 K and diffraction patterns were again collected every 5 min. Fitting the Avrami equation gives τ ≃ 230 min and τ_e_ ≃ 510 min. As a final investigation of the intermediate-temperature regime, a specimen was annealed for 10 h at 698 K. After 10 h of annealing only the first signs of decomposition are visible. Both the α-U peaks and the U_2_Mo peak start to grow, while the γ-UMo peak decreases. The collected data were not sufficient for an Avrami fit and therefore the beginning and end of peak growth could not be determined. Hence, the data given for 698 K only give an estimate for the beginning of phase decomposition.

Table 4 ▸ summarizes the results derived from the Avrami fit for all investigated temperatures in the high- and intermediate-temperature regimes. It is noteworthy that, for all Avrami fits, *n* ≃ 3 within the margin of error, whereas for a three-dimensional nucleation and growth process *n* ≥ 3. For a zero nucleation rate it is *n* = 3 and for a decreasing nucleation rate it is 3 < *n* < 4. Therefore, the results suggest either a zero or a decreasing nucleation rate (Christian, 2002 ▸).

### 5.2. Crystallographic phase analysis *via* Rietveld refinement   

#### 5.2.1. High-temperature reactions (748 and 773 K)   

After annealing for 3 h at 748 K, already a large part of the initial γ-UMo is transformed. Besides α-U, another product at the beginning of decomposition during high-temperature reactions is a molybdenum-enriched phase γ-UMo-b. As expected for high-temperature reactions, the decomposition started with forming α-U and enriched γ-UMo-b. This can be seen by the relatively high content of these two phases. Furthermore, some U_2_Mo has already formed.

The two γ-UMo peaks are very close together, and therefore distinguishing between these two phases is prone to error. Hence, there is the possibility that some calculated content of γ-UMo-a belongs to γ-UMo-b, or *vice versa*.

It is significant that the amount of γ-UMo-a drops fast along with the fast growth of α-U. The α-U growth already approaches its saturation before 24 h. The amount of γ-UMo-b increases very fast but then decreases fast shortly after reaching its maximum. This can be explained by the fact that a gradually increasing enrichment of the γ-UMo-b phase in Mo leads to the formation of U_2_Mo at the cost of γ-UMo-b.

Table 5 ▸ summarizes the calculated lattice constants for the two γ-UMo, U_2_Mo and α-U phases for each measurement. The behaviour of γ-UMo-b shows the molybdenum enrichment with time. The longer the specimen was annealed, the smaller are the lattice parameters, and hence the higher is the molybdenum content in γ-UMo-b.

The calculated lattice parameters for α-U show expanded parameters *a* and *c*, together with a contracted parameter *b*, compared with a pure α-U phase. This indicates that the present uranium phase is the distorted phase α′-U. The lattice parameters for γ-UMo, α-U and the suggested α-U phase are in good agreement with results previously obtained by Palancher *et al.* (2012 ▸) for U–8 wt%Mo samples. It should be noted that Palancher and co-workers investigated UMo/Al(Si) nuclear fuel plates rather than pure UMo samples.

At 773 K, similar behaviour to the reactions at 748 K is expected because both temperatures belong to the high-temperature reaction regime. However, a noticeable difference can be seen between samples A-748K3h and A-773K3h. Owing to the very sharp peak of the γ-UMo Bragg reflection, small changes to the peak-shape parameters have a huge effect on the crystallographic composition without influencing the quality of the fit in a significant manner. Moreover, it was not possible to refine two different γ-UMo phases inside these diffractograms. Therefore, no quantitative information is given for sample A-773K3h apart from the knowledge that decomposition has already started.

The calculated lattice parameters summarized in Table 5 ▸ show no distinctive features except for the missing second γ-UMo phase in sample A-773K3h. Again, the distortion of the pure α-U phases suggests the presence of α′-U rather than α-U.

#### 5.2.2. Intermediate-temperature reactions (673, 698 and 723 K)   

The decomposition takes place differently in the intermediate-temperature regime compared with the high-temperature regime. While at 723 K a noticeable decomposition already took place after 3 h of annealing, at the lower temperatures the beginning of phase decomposition is delayed. And not only is the onset of phase decomposition delayed, but also the transformation itself takes place at a much slower rate than at higher temperatures.

At 723 K, the scan of A-723K3h shows that the decomposition has already started. Rietveld analysis of the diffraction data for this sample was difficult, owing to an uneven background and UC inclusions at the position of the beam spot in the experiment. Since X-ray measurements were only performed on small areas (0.2 × 0.2 mm) on the samples, any inclusions or grain structure present strongly influenced the results for these measurements. Therefore, the results for the amount of α-U and U_2_Mo in the A-723K3h sample differ from measurements where the grain size is negligible.

Since the intermediate-temperature reactions contain both the low- and high-temperature reactions, and because 723 K is at the top of the intermediate-temperature regime, a comparable result to the high-temperature regime was expected for decomposition. The irregularity in the decrease of the γ-UMo peaks and the increase of U_2_Mo are again due to overlapping peaks of these phases.

Small lattice parameters are apparent for γ-UMo-b after 16 h of heat treatment (Table 5 ▸), where the other lattice parameters for this measurement show no anomalies. Moreover, the lattice constants for the other measurements show the expected behaviour. The α-U phase is still α′-U.

The nucleation process is very sluggish for temperatures of 698 K or less and hence the decomposition starts much later than for higher temperatures. After 3 h of annealing there is still no evidence for decomposition. The composition of sample A-698K3h shown in Table 6 ▸ is similar to that of sample A-initial. Therefore, after 3 h of heat treatment, the sample still consists of one highly homogeneous γ-UMo phase.

These data suggest that the nucleation period is somewhere between 3 and 6 h. A-698K6h shows a slight decomposition of the γ-UMo phase compared with A-698K3h. The results for A-698K24h reveal differences between the progress of decomposition in the high- and intermediate-temperature regimes. And not only the nucleation but also the decomposition itself is more sluggish: after 24 h, a large amount of γ-UMo is still left. A striking aspect is that the amount of U_2_Mo is much lower compared with the results obtained after annealing at 723–773 K, while the amount of α-U is only slightly lower. A retarded decomposition explains the difference in the amount of α-U but not the difference in the U_2_Mo content. Although the reactions in the intermediate-temperature regime start with a cellular reaction as in the high-temperature regime, they take a different course because of the low-temperature reactions and the formation of a Widmannstätten α structure (Repas *et al.*, 1964 ▸). These differences explain the rather high amount of α-U together with the rather low amount of U_2_Mo. The amount of UC is higher than for most of the samples and can again be explained by the measurement conditions with X-rays, where the lack of a homogeneous distribution of the carbides causes disproportionally high amounts of UC. The lattice constants listed in Table 5 ▸ show no distinctive features and the suggested α-U phase is still α′-U.

Owing to the sluggish nucleation and hence the retarded decomposition at lower temperatures, only two samples were annealed at 673 K, for 24 h (A-673K24h) and 48 h (A-673K48h).

The specimen A-673K24h shows only a slight decomposition and therefore it was not possible to fit the neutron diffraction data with a second γ-UMo phase. The most distinctive features compared with the higher temperatures can be seen in the crystallographic compositions shown in Table 6 ▸. The increase in the rising phases is not only retarded but also starts with U_2_Mo rather than with α-U. This is due to the low-temperature reactions, where the decomposition is initiated by the formation of U_2_Mo without the presence of α-U, whereas the latter is subsequently precipitated. Since 673 K is still in the intermediate-temperature regime, the high-temperature reactions occur as well and ensure the formation of α-U at the very beginning of decomposition. After 48 h, only half of the initial γ-UMo has decomposed into equal parts of α-U and U_2_Mo, as well as some enriched γ-UMo.

The lattice parameters for the 673 K measurements in Table 5 ▸ are in good agreement with the results for the higher temperatures and show no distinctive features. Therefore, the suggested phase for α-U is still α′-U. Previous results by Palancher and co-workers found α′′-U in samples with about 7 wt% molybdenum after annealing at temperatures of 698 K or less, and measurements on U–8 wt%Mo samples delivered an α′-U crystal structure (Palancher *et al.*, 2012 ▸, 2013 ▸). This might be due to the formation of phases including U and Al in the dispersed UMo/Al samples examined by Palancher and co-workers. Hence, relatively more Mo remains inside the UMo kernels, which might lead to the formation of molybdenum-enriched α′′ instead of α′. The relationship between the lattice parameters and the crystallographic structure was discussed in their reports. The same behaviour of α-U for temperatures below 698 K was observed in this work. Moreover, refining the crystal structure of α-U, which is the same for the distortion α′-U but not for α′′-U, was successful and caused no problems. This leads us to conclude that the specimens annealed at 698 and 673 K still contain α′-U rather than α′′-U.

The final agreement factors of the Rietvield refinements, which define the quality of the fit, are shown in Table 7 ▸. A striking aspect is that the refinements of the X-ray patterns have larger χ^2^ values, which is explained by one major factor. Although *R*_exp_ decreases as the number of counts collected for the diffraction pattern is increased, the difference between *R*_exp_ and *R*_wp_ becomes larger. Hence, χ^2^ is worse even though the model fitted to the diffraction pattern is improved. The reason for this is that, for diffraction patterns with a very large number of counts, even minor imperfections in the peak shape or peak position and unmodelled features of the background can make it impossible to obtain small values for *R*_wp_ and hence for χ^2^. Such imperfections can be seen in the diffraction patterns collected by XRD, where the scattering at the aperture of the experiment induced small peaks in the diffraction pattern at 0.24 Å^−1^ which are not included in the fit.

Besides the agreement factors, the difference between the data and the calculated pattern, *Y*_obs_ − *Y*_calc_, can be taken as an indicator of the quality of the fit. This shows that the models obtained for the X-ray patterns are not worse than those obtained for the neutron patterns, as suggested by the final agreement factors. The discrepancy is well explained by the fact of a much higher number of counts for the measurements with X-rays.

### 5.3. Isothermal transformation diagram for U–8 wt%Mo   

Comparing the growth curves with the data on the crystallographic compositions of the annealed samples shows that, after the growth of the α-U peak has stopped as described by the S-shaped Avrami curve, the peak intensity keeps growing with a linear-like behaviour and much more slowly compared with the S-shaped growth. The linear-like growth slowly approaches the saturation of this phase.

**Figure 6:**
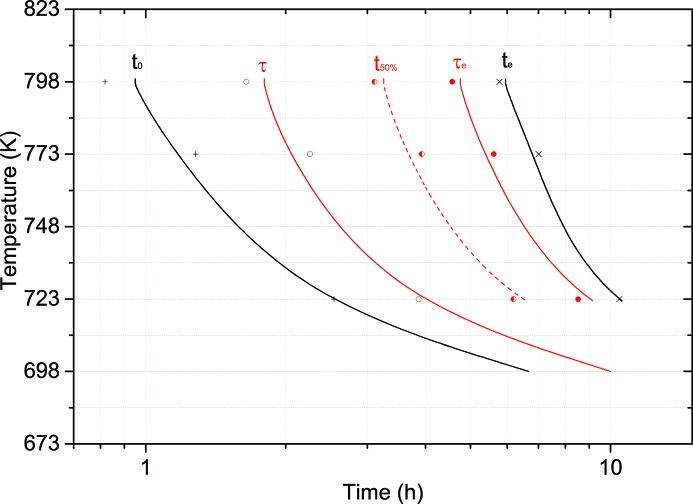
Isothermal transformation curves for α-U phase growth as determined by the Avrami equation.

Fig. 6 ▸ shows the α-U phase growth according to Table 4 ▸ and is described by the Avrami equation. Since α-U is the first product of the transformation, Fig. 6 ▸ describes the beginning of phase decomposition along with detailed information on α-U growth.

**Figure 7:**
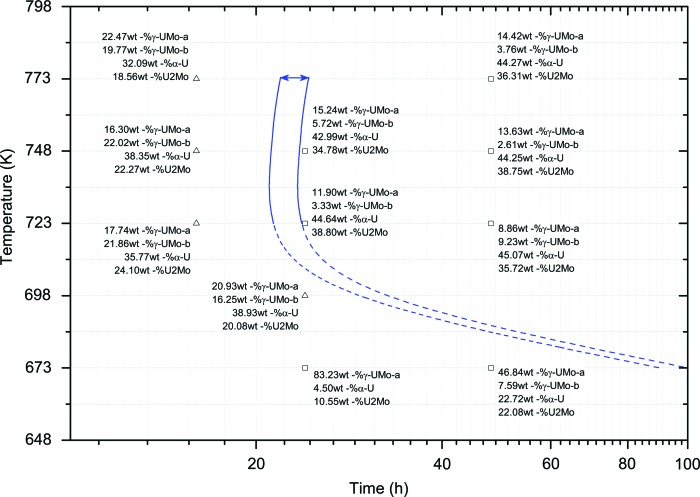
The measuring points and their determined crystallographic composition, along with the isothermal transformation region describing the beginning of the linear-like increase of U_2_Mo.

In Fig. 7 ▸ the crystallographic phase compositions at different measurement points are displayed. The data suggest that the S-shaped growths of U_2_Mo and α-U start together but increase differently. The growth of U_2_Mo takes much longer. Comparing the data obtained for measurements on samples annealed for 24 and 48 h, respectively, for temperatures between 723 and 748 K suggests that U_2_Mo keeps growing with a linear-like increase after the S-shaped growth. This is the same behaviour as observed for α-U. Therefore, the blue area drawn in Fig. 7 ▸ indicates the beginning of the linear-like increase of U_2_Mo and the approach to the end of the Avrami-like phase decomposition.

Different definitions for the start and end of phase decomposition define the positioning of the C-shaped curves in a TTT diagram. While earlier work used the first signs of phase decomposition to define transition curves, a definition *via* growth curves can be found in the current literature. The Avrami fit allows us to extract the time for any fraction of transformed phases from the data obtained during *in situ* annealing.

Comparing the growth curves with data on the crystallographic composition of the annealed samples shows that, after the fast growth of a peak has stopped at τ_e_, the peaks keep growing with a linear-like behaviour and much more slowly than the fast growth. The latter statement is supported by the peak-growth behaviour observed at 773 K discussed in §5.1, where not only fast growth was observed but also the growth for *t* > τ_e_. This linear-like growth of a peak slowly approaches the saturation of this phase. The time when the fast growth reaches the linear-like growth could not be measured exactly, since the measurements were not performed over such a long period for 773 K, so it was calculated using the Avrami equation for each temperature.

Hence, the following information for the TTT diagram is obtained by *in situ* annealing studies:

(i) *t*_0_: the first signs of phase decomposition, *i.e.* 1% of the α-U peak fast growth;

(ii) τ: the start of phase decomposition, 10% of fast peak growth according to Avrami;

(iii) *t*_50%_: 50% of the fast growth is reached at the point of inflection;

(iv) τ_e_: the end of phase decomposition, 90% of fast peak growth according to Avrami;

(v) *t*_e_: the start of the linear-like increase after fast growth is finished, *i.e.* 99% of the α-U peak fast growth.

The contribution of *in situ* annealing studies to the isothermal transformation diagram is depicted in Fig. 6 ▸. Plotted are the above-described times describing the fast growth of α-U and hence the beginning of phase decomposition, along with detailed information on α-U growth.

The information obtained from crystallographic phase analysis contributes less significantly to the C curves for the beginning of phase decomposition in the TTT diagram. This has its reasons in the wide distribution of the measuring points in time. Although measurements at 3 and 6 h at a certain temperature were not sufficient to observe the fast growth of peaks, measuring points between *t*_0_ and *t*_e_ can still be used to complement the fast-growth curves and support the results obtained from the *in situ* annealing studies.

Analysing severely decomposed material, on the other hand, gives insight into the stage of phase decomposition. The data suggest that the fast growths for U_2_Mo and α-U start together but increase differently. The fast growth of U_2_Mo takes much longer. Comparing the data obtained for measurements on samples annealed for 24 and 48 h, respectively, at temperatures between 723 and 748 K suggests that U_2_Mo keeps growing with a linear-like increase after the fast growth. Therefore, the data indicate the final stage of the U_2_Mo fast growth and thereby the points after which the content of the transformation products increases only slightly with time. An exact determination of this transformation curve is not possible because measurements were only performed for 16 and 24 h, and not somewhere in between. Thus, a C-shaped area describing the final stage of U_2_Mo fast growth and the approach of the end of phase decomposition can be estimated. Fig. 7 ▸ shows the isothermal transformation curve describing the final stage of U_2_Mo fast growth, along with the measuring points and their determined crystallographic compositions. The dashed part of the curve thus describes an estimation, since no sample was prepared with annealing at 698 K for 48 h or for times greater than 48 h at 673 K.

**Figure 8:**
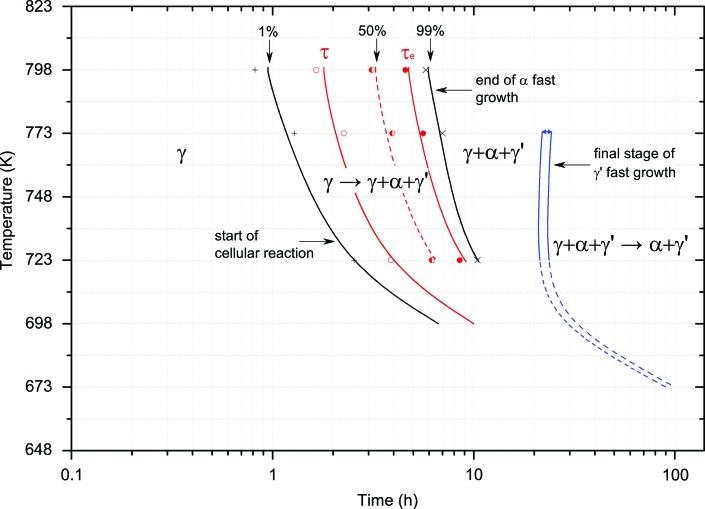
Isothermal transformation diagram of U–8 wt%Mo.

The final isothermal transformation diagram in Fig. 8 ▸ comprises the curves from Figs. 6 ▸ and 7 ▸. The diagram shows the start of the cellular reaction where γ-UMo starts to transform into α-U and U_2_Mo, the end of the α-U phase fast growth, the final stage of the U_2_Mo fast growth, and the region where the remaining γ-UMo slowly vanishes as the α-U and U_2_Mo phases increase with a linear-like behaviour.

**Figure 9:**
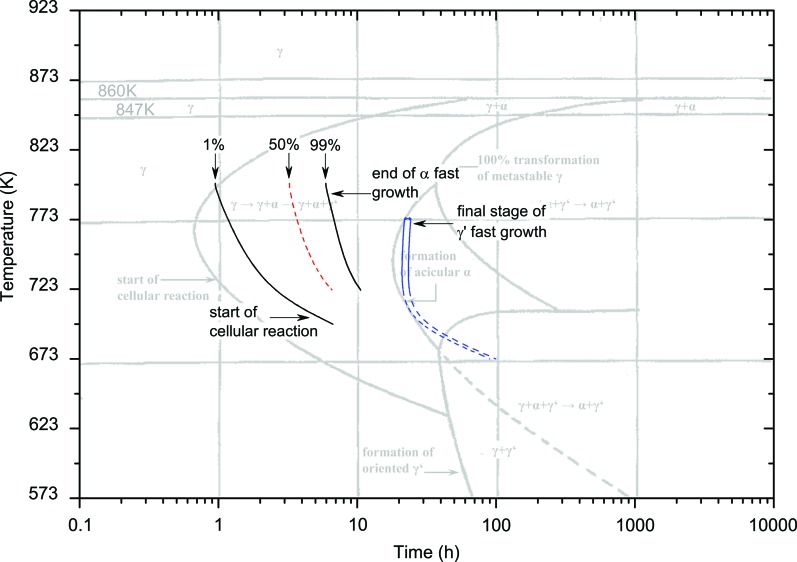
Isothermal transformation curves obtained in this work compared with the diagram obtained by Repas *et al.* (1964 ▸) (light grey). (Reprinted with the permission of ASM International. All rights reserved.)

The results of this work can be compared with the previous results found in the literature. Fig. 9 ▸ shows the isothermal transformation curves obtained in this work and the TTT diagram for U–8 wt%Mo proposed by Repas *et al.* (1964 ▸). The latter work obtained the curves by preparing a grid of samples examined *via* metallurgical methods and dilatometric, microhardness and XRD data. Thus, an exact definition of the start of phase decomposition was not obvious. Moreover, the number of measurement points, and therefore the density of the measurement grid, was not given.

## 6. Conclusion   

This work complements previous similar experiments considering neutron diffraction of UMo/Al systems exposed to elevated temperatures between 673 and 748 K and with annealing times between 2 and 52 h (Palancher *et al.*, 2013 ▸). In the present work, a wider range of annealing temperatures and a more precise stepping in annealing time were used in order to provide a more detailed investigation of the growth kinetics.

The isothermal transformation curves obtained in this work contain detailed information on the fast growth of α-U. The data show that fast growth for U_2_Mo is much slower than that for α-U. Despite suggestions found in the literature where first the α-U precipitates and then U_2_Mo starts to grow later, the diffraction data presented here clearly reveal that both transformation products start to grow simultaneously. The C-shaped area of the diagram at long annealing times describes the end of the U_2_Mo fast growth and the approach of the end of phase decomposition. A 100% transformation of metastable γ-UMo could not be seen within 48 h of annealing at any temperature.

## Figures and Tables

**Figure 2:**
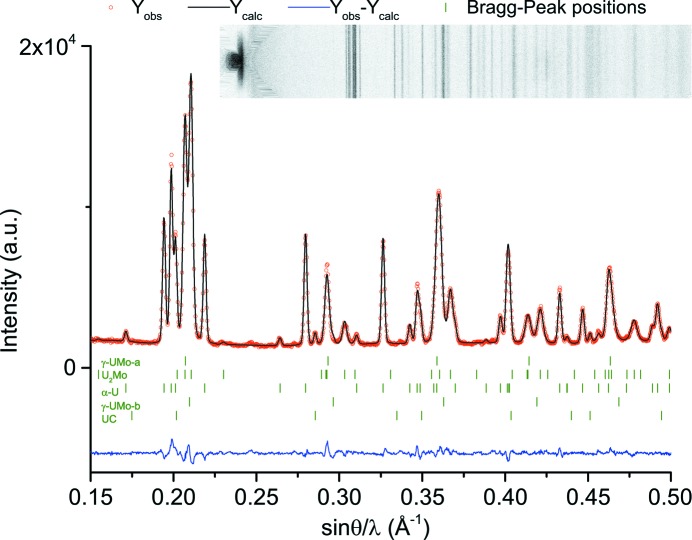
The neutron diffraction pattern for the sample A-748K48h with the diffraction image (inset). Red circles indicate the measured data, green dashes the Bragg peak positions, the black line the calculated pattern, and the blue line the difference between the calculated pattern and the measured data.

**Table 1:** Samples analysed by neutron diffraction (crystallographic phase analysis)

Sample	*T*_anneal_ (K)	*t*_anneal_ (h)
A-initial		
A-673K24h	673	24
A-673K48h	673	48
A-698K3h	698	3
A-698K6h	698	6
A-723K24h	723	24
A-723K48h	723	48
A-748K6h	748	6
A-748K24h	748	24
A-748K48h	748	48
A-773K3h	773	3
A-773K48h	773	48

**Table 2:** Samples analysed by X-ray diffraction (crystallographic phase analysis)

Sample	*T*_anneal_ (K)	*t*_anneal_ (h)
A-698K24h	698	24
A-723K3h	723	3
A-723K16h	723	16
A-748K3h	748	3
A-748K16h	748	16
A-773K6h	773	6
A-773K16h	773	16

**Table 3:** Samples analysed by neutron diffraction (*in situ* annealing studies)

Sample	*T*_anneal_ (K)	*t*_anneal_ (h)
A-698Kinsitu	698	0–10
A-723Kinsitu	723	0–10
A-773Kinsitu	773	0–7
A-773K16h	773	16–36
A-798Kinsitu	798	0–4.5

**Table 4:** Overview of α-U growth depending on annealing temperature (K) and time (min) *t* and τ indicate the weight fraction of α-U after the corresponding annealing time. The data for 698 K are estimated since the measurement time was insufficient to collect satisfactory data.

Annealing temperature	*t*_0_ = 1%	τ = 10%	*t*_50%_ = 50%	τ_e_ = 90%	*t*_e_ = 99%
798	49	99	186	274	346
773	77	135	236	336	420
723	152	232	371	511	627
698	∼360–420	∼540–660			

**Table 5:** Lattice constants (Å) of the phases γ-UMo-a, γ-UMo-b, α-U (α′-U, α′′-U) and U_2_Mo for the isothermal studies

		γ-UMo-a	γ-UMo-b	U_2_Mo		α-U			
Examining radiation	Annealing temperature and duration	*a* = *b* = *c*	*a* = *b* = *c*	*a* = *b*	*c*	*a*	*b*	*c*	α-U phase
Neutrons		3.427							
Neutrons	673 K, 24 h	3.427		3.436	9.923	2.866	5.867	4.955	α′
Neutrons	673 K, 48 h	3.427	3.387	3.426	9.931	2.867	5.851	4.956	α′
Neutrons	698 K, 3 h	3.427							
Neutrons	698 K, 6 h	3.428		3.448	9.893	2.867	5.841	4.967	α′
X-rays	698 K, 24 h	3.418	3.376	3.407	9.911	2.863	5.837	4.952	α′
X-rays	723 K, 3 h	3.417	3.393	3.400	9.890	2.864	5.824	4.958	α′
X-rays	723 K, 16 h	3.405	3.358	3.414	9.891	2.861	5.831	4.950	α′
Neutrons	723 K, 24 h	3.403	3.370	3.415	9.901	2.868	5.840	4.971	α′
Neutrons	723 K, 48 h	3.407	3.379	3.414	9.925	2.867	5.841	4.968	α′
X-rays	748 K, 3 h	3.419	3.389	3.400	9.958	2.862	5.824	4.961	α′
Neutrons	748 K, 6 h	3.426	3.395	3.407	9.993	2.869	5.839	4.971	α′
X-rays	748 K, 16 h	3.400	3.362	3.406	9.909	2.862	5.826	4.960	α′
Neutrons	748 K, 24 h	3.410	3.373	3.417	9.904	2.867	5.837	4.971	α′
Neutrons	748 K, 48 h	3.412	3.374	3.419	9.893	2.867	5.839	4.971	α′
Neutrons	773 K, 3 h	3.426		3.425	10.139	2.869	5.840	4.978	α′
X-rays	773 K, 6 h	3.399	3.383	3.406	9.911	2.862	5.822	4.961	α′
X-rays	773 K, 16 h	3.405	3.362	3.408	9.882	2.861	5.823	4.960	α′
Neutrons	773 K, 48 h	3.415	3.380	3.416	9.888	2.867	5.835	4.972	α′

**Table 6:** Crystallographic composition of U–8 wt%Mo for isothermal studies for different post-manufacturing thermal treatments (weight fraction, wt%)

Examining radiation	Annealing temperature and duration	γ-UMo-a	γ-UMo-b	U_2_Mo	α-U	UC
Neutrons		98.93				1.07
Neutrons	673 K, 24 h	83.23		10.55	4.50	1.72
Neutrons	673 K, 48 h	46.84	7.59	22.08	22.72	0.76
Neutrons	698 K, 3 h	99.06				0.94
Neutrons	698 K, 6 h	95.12		1.34	2.51	1.04
X-rays	698 K, 24 h	20.93	16.25	20.08	38.93	3.82
X-rays	723 K, 3 h	56.84	31.23	0.01	6.29	5.64
X-rays	723 K, 16 h	17.74	21.86	24.10	35.77	0.53
Neutrons	723 K, 24 h	11.90	3.33	38.80	44.64	1.32
Neutrons	723 K, 48 h	8.86	9.23	35.72	45.07	1.12
X-rays	748 K, 3 h	54.67	20.32	5.56	18.38	1.07
Neutrons	748 K, 6 h	22.44	27.07	19.42	29.93	1.15
X-rays	748 K, 16 h	16.30	22.02	22.27	38.35	1.06
Neutrons	748 K, 24 h	15.24	5.72	34.78	42.99	1.28
Neutrons	748 K, 48 h	13.63	2.61	38.75	44.25	0.76
Neutrons	773 K, 3 h					
X-rays	773 K, 6 h	22.47	26.92	13.47	36.22	0.92
X-rays	773 K, 16 h	19.77	29.25	18.56	32.09	0.33
Neutrons	773 K, 48 h	14.42	3.76	36.31	44.27	1.23

**Table 7:** Final agreement factors (%) between calculated and measured data obtained by Rietveld refinement of the diffraction patterns for the isothermal studies

Examining radiation	Annealing temperature and duration	*R*_p_	*R*_wp_	*R*_exp_	χ^2^
Neutrons		11.0	8.95	3.7	7.06
Neutrons	673 K, 24 h	12.7	14.0	3.15	19.7
Neutrons	673 K, 48 h	6.75	6.99	3.46	4.09
Neutrons	698 K, 3 h	8.68	7.07	2.51	9.17
Neutrons	698 K, 6 h	10.9	8.45	3.15	7.26
X-rays	698 K, 24 h	8.85	9.61	4.70	4.39
X-rays	723 K, 3 h	33.3	26.5	14.6	3.29
X-rays	723 K, 16 h	5.25	7.11	1.21	34.7
Neutrons	723 K, 24 h	6.91	7.40	3.25	5.22
Neutrons	723 K, 48 h	7.25	8.03	3.90	4.24
X-rays	748 K, 3 h	6.50	7.66	2.83	7.45
Neutrons	748 K, 6 h	8.18	7.92	4.39	3.25
X-rays	748 K, 16 h	4.78	6.42	1.21	28.3
Neutrons	748 K, 24 h	7.47	8.08	3.34	5.92
Neutrons	748 K, 48 h	7.48	7.75	4.02	3.77
Neutrons	773 K, 3 h	15.1	13.6	3.05	20.0
X-rays	773 K, 6 h	5.03	6.43	1.19	29.5
X-rays	773 K, 16 h	4.66	6.13	1.17	27.7
Neutrons	773 K, 48 h	6.76	7.21	3.86	3.62
